# Owl-like plaques of the Copper Age and the involvement of children

**DOI:** 10.1038/s41598-022-23530-0

**Published:** 2022-12-01

**Authors:** Juan J. Negro, Guillermo Blanco, Eduardo Rodríguez-Rodríguez, Víctor M. Díaz Núñez de Arenas

**Affiliations:** 1 Department of Evolutionary Ecology, Estación Biológica de Doñana, CSIC, Avda. Americo Vespucio 26, 41092 Sevilla, Spain; 2 Department of Evolutionary Ecology, Museo Nacional de Ciencias Naturales, CSIC, José Gutiérrez Abascal 2, 28006 Madrid, Spain; 3 Department of Integrated Sciences, Faculty of Experimental Sciences, Avda de Las Fuerzas Armadas S/N, Universidad de Huelva, 21007 Huelva, Spain; 4 Department of Art History, Faculty of Geography and History, Universidad Complutense de Madrid, Profesor Aranguren, S/N. Ciudad Universitaria, 28040 Madrid, Spain

**Keywords:** Zoology, Archaeology

## Abstract

In the Copper Age, slate engraved plaques were produced massively in the southwestern corner of the Iberian Peninsula. Researchers have speculated about the function of these palm-sized stone objects for more than a century, although most have favored the idea that they represented goddesses, and served ritual purposes. The plaques are engraved with different designs of varying complexity. In some of them, the ones sporting two large frontal eyes, we clearly see owls modelled after two species present in the area: the little owl (*Athene noctua*), and the long-eared owl (*Asio otus*). These two species, living in semi-open habitats, were possibly the most abundant owls around the human settlements and surrounding cultivated fields of the Chalcolithic period. People must have been aware of the owl presence and possibly interacted with them. Why owls but no other animals have been the models may relate to the fact they are the most anthropomorphic of all animals, with large frontally-placed eyes in their enormous heads. In the iconography, owls are systematically represented, even today, with their two eyes staring at the observer, as opposed to the lateral view used for any other animal. Additionally, slate is one of the commonest surface rocks in southwestern Iberia, and it provides a blank canvas for engraving lines using pointed tools made of flint, quartz or copper. The way slates exfoliate makes easy to craft owl-looking plaques. To silhouette animals other than owls in a recognizable way would request extra carving abilities and specific tools. Plaque manufacture and design were simple and did not demand high skills nor intensive labor as demonstrated in replication experiments. Owl engravings could have been executed by youngsters, as they resemble owls painted today by elementary school students. This also suggests that schematic drawings are universal and timeless. We propose that the owl-like slate plaques are the remains of a set of objects used in both playful activities and in ritual ceremonies. The actual engraving of the plaques may have been part of the game. Owlish slate plaques were often perforated twice at the top. We interpret this as insertion points for actual bird feathers added to the plaques, right at the place where tufts emerge in live owls. The frontier among play and ritual is diffuse in liminal societies and there is no contradiction in playing with animal-like toys and, at some point, using them as offerings as part of community rituals related, for instance, to the colossal megalithic tombs so characteristic of the Copper Age.

## Introduction

**Figure 1:**
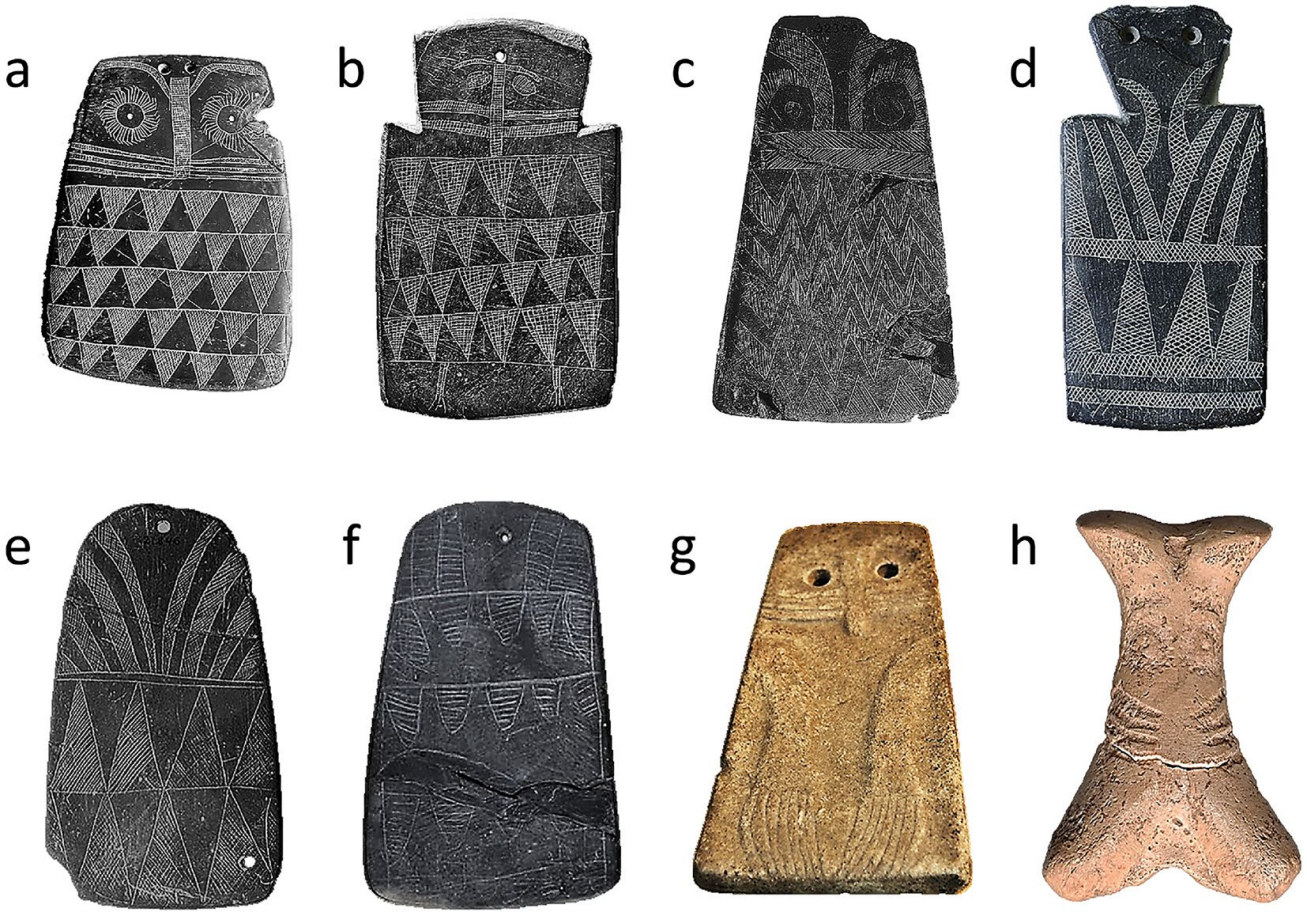
Selected plaques engraved in the Chalcolithic period in the southwestern portion of the Iberian Peninsula. These examples provide a glimpse of the different typologies and materials used (not at scale). (**a**) Slate plaque from Cerro de las Cabezas, Valencina de la Concepción, Sevilla, Spain (Museo Arqueológico de Sevilla, REP25837). (**b**) Slate plaque from anta do Curral da Antinha, Arraiolos, Evora, Portugal (Museu Nacional de Arqueologia de Portugal [MNA], Lisboa, 2003.37.1). (**c**) Slate plaque from anta 1 da Farisoa, São Marcos do Campo, Reguengos de Monsaraz, Evora, Portugal (MNA, Lisboa, 32300). (**d**) Slate plaque with carved head from anta da Marquesa, Marvão, Portalegre, Portugal (MNA, Lisboa, 8195). (**e**) Slate plaque from anta Grande do Olival da Pega, Reguengos de Monsaraz, Evora, Portugal (MNA, Lisboa, 985.45.21). (**f**) Slate plaque from anta Grande do Olival da Pega, Reguengos de Monsaraz, Evora, Portugal (MNA, Lisboa, 985.45.15). (**g**) Sandstone plaque from anta da Horta, Alter do Chão, Portoalegre, Alentejo, Portugal (Museu da Coudelaria de Alter, Portugal, AH 197). (**h**) Oculated idol carved on proximal phalanx of ungulate from Huerta de Dios, Casas de Reina, Badajoz, Spain (Museo Arqueológico Provincial de Badajoz, 11425).

The engraved slate plaques of southwestern Iberia were crafted in a relatively narrow time window about 5500 to 4750 years BP^1^, and rank among the most emblematic and unique objects of the Chalcolithic/Copper Age cultural period (Fig. 1). These palm-sized plaques were engraved with geometric patterns and often had a “head” with two round circles generally described as eyes^2^ and a “body” below^3^. Most of them had one or two perforations at the top of the head purportedly used to pass a string^2^. In addition to the flat slate plaques, similar objects carved in sandstone or in mammal bones such as horse phalanges, have been found, also exhibiting a pair of eyes on one of the extremes (Fig. 1). About 4000 plaques have been located so far^4^, many on communal megalithic graves such as tholos, but also in pits. Today, they are dispersed in a multitude of archaeological museums and ethnographic collections, mainly in Spain and Portugal^2^.

The first discovered plaques were studied at the onset of systematic archaeology in the late nineteenth century in Portugal^2^, but research and curiosity has persisted for more than a century until today. The interpretation of their origin and function has varied along this period. Quite early, some scholars^5^ noticed similarities with predynastic slate palettes from Egypt and suggested an eastern origin for the style, even though the main raw material were the abundant local slates (Spain is still the leading producer of roofing slates, with 90% of the world production^6^). Early prehistorians held the hypothesis the plaques served a religious and/or symbolic purpose (e.g., Vasconcelos^7^). A few years later, Correia^8^ interpreted the plaques as anthropomorphic idols representing a female goddess, as with the female divinities found all along the Mediterranean^9,10^. An alternative is later provided by Frankowski^11^, who argued that the plaques were not goddesses but representations of the dead themselves.

**Figure 2:**
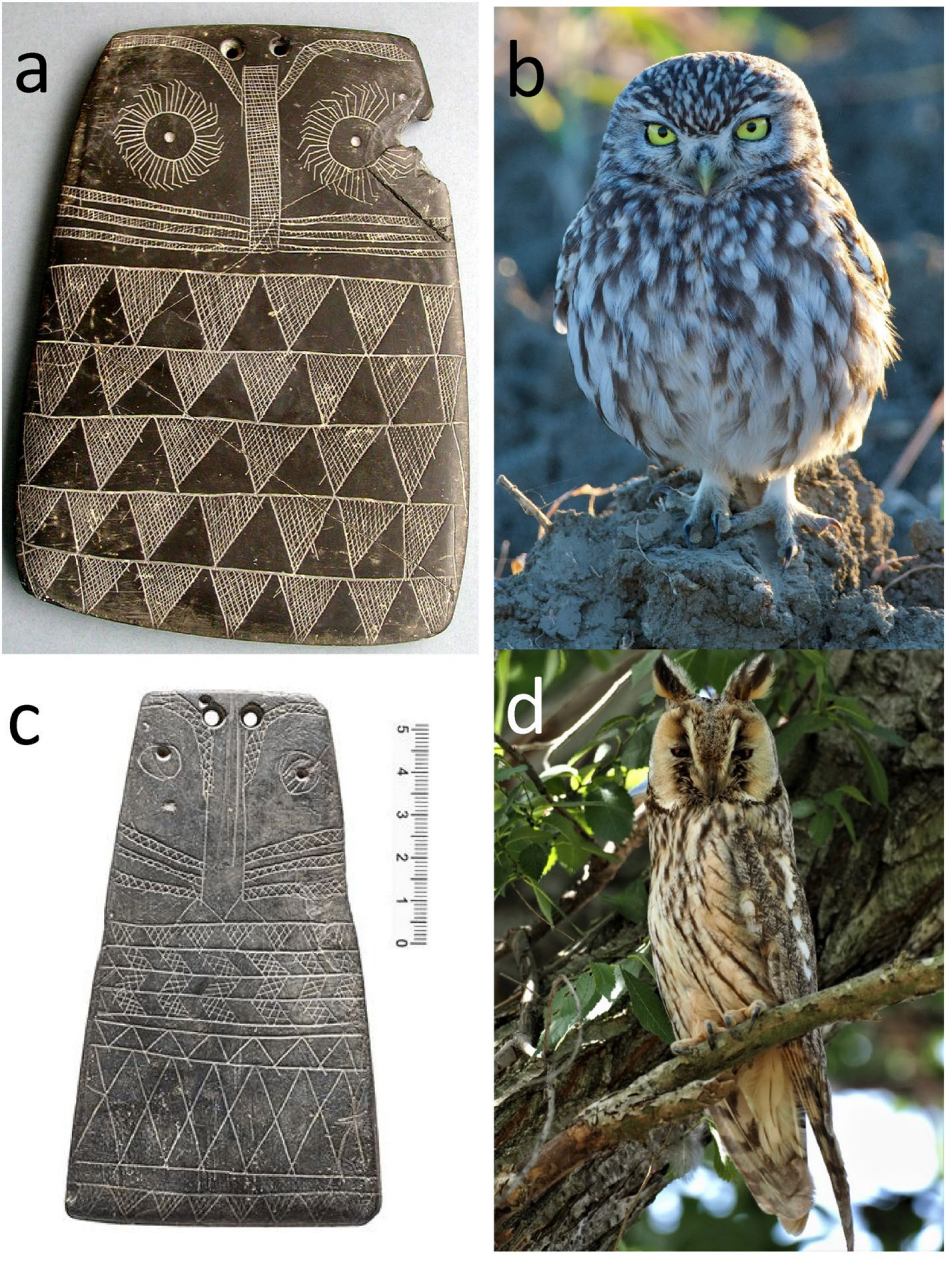
Upper left (**a**), Engraved slate plaque found in Cerro de la Cabeza, Valencina de la Concepción, Sevilla, España (Museo Arqueológico de Sevilla, Spain), representative of the zoomorphic type recognized by Lillios^2^. Upper right (**b**), wild little owl (*Athene noctua*) pictured in an agricultural area in Valencina de la Concepción in 2021. Bottom left (**c**), slate plaque from Mértola, Alentejo, Portugal (MNA, Lisboa, 2006.370.1). The orifices on the upper part may have been used to insert actual feathers. On the right (**d**), long-eared owl (*Asio otus*) with erected ear tufts, pictured in Valencina de la Concepción in 2021. Credits little and long-eared owls: Juan J. Negro.

Marija Gimbutas^9,10^ stated that the Iberian plaques were representations of a Mother Goddess, sometimes featured as an owl. Although the frontal view of an owl is utterly evident in many plaques (Fig. 2), Gimbutas’ identification has hardly ever been cited in recent years. In the latest reviews^12,13^, the slates are liberally called anthropomorphic idols, implying their use in rituals, and the resemblance to owls and its implications are overlooked.

In some of the engraved plaques we see owls depicted frontally and staring at the observer, even if a cartoon-like owl, and as if conceived by a child. In fact, the hypotheses that we posit are two: (1) the slate plates of the Chalcolithic of Iberia and related objects with two frontal eyes are inspired in actual owls, and represent them either figuratively (the biomorphic ones) or in abstract form; (2) contrary to previous interpretations ascribing a highly ritualistic and deep significance to these objects (see references above), we suggest that the commonness of the material used and their basic and fast manufacture—as demonstrated by Lillios^2^ and Thomas^3^—point to their crafting and use by young members of the community, possibly as dolls, toys, drawings or amulets. The use of the plaques as recreational objects is, in any case, not mutually exclusive with other uses, as many ended up as possible votive elements in burials. As reviewed by Langley and Litster^14^, or Renfrew^15^, objects used to play, or functional such as pottery^16,17^ may overlap with items used in adult rituals. Maicas^18^ acknowledged these dual purposes remembering that in Chalcolithic societies recreational and ritual functions were not necessarily dissociated.

This approach assumes that the owl-like objects may be the archaeological trace of playful and learning activities carried out by youngsters, who were possibly the largest demographic group^14^, and therefore a fundamental part of Chalcolithic society^19^. Ignoring this fact may have led to identifying certain finds as symbolic or ritual when, quite simply, they are the trace of playful behaviors or learning activities. So far, the earlier set of toys may be the one found at the Siberian Upper Paleolithic site of Mal’ta, which included human, bird and other animal figurines^20^. Also in Siberia a human figurine and several animals, have been found at baby graves, dated 4500 years BP in the Bronze Age^21^. Whether or not the Chalcolithic slate plaques can be considered toys will remain speculative, but it is a fact that in ethnographic examples worldwide, many artisan practices, including pottery^16,17,22^, are learnt in childhood.

Last, we aimed to identify the owls depicted in the plaques to species. There were and still are few species of owls in Iberia and they differ in some recognizable ways. We will use these traits to make a proposal regarding the owl species depicted in at least the most figurative of all plaques. Identifying owl species can help to understand the circumstances and motivations for their depiction, as the ecology of the different species tells us about the context in which interaction with humans occurred, and their connections to the attributions and potential symbolism associated to them. In addition to this, we review other evidences that support the notion that owls were used as models in the Chalcolithic, acknowledging at the same time that numerous plaques cannot be directly ascribed to owls and may deserve alternative explanations.

## Material and methods

**Figure 3:**
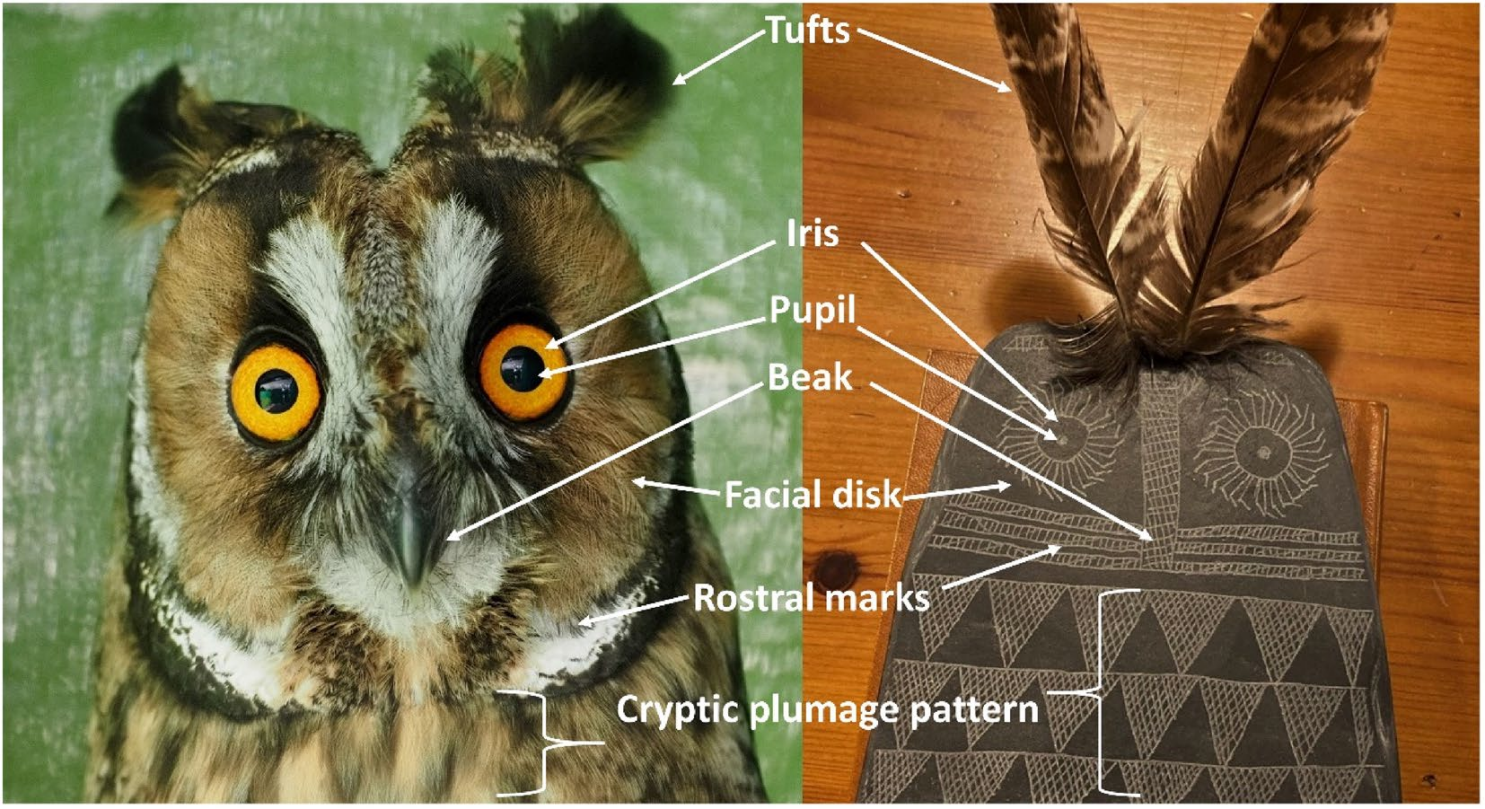
Correspondence of the main traits of facial and ventral areas of a long-eared owl and a feather-decorated replica of the Valencina slate plaque. Credits: Juan J. Negro.

Our sources are mainly bibliographic, both concerning iconography of owls through the ages^23^, and specifically concerning pictures and drawings of slate plaques and other artifacts. In a few instances, the authors obtained photographs of some plaques from public collections, such as Museo Arqueológico Nacional, Madrid (MAN) and Museo Arqueológico Provincial de Badajoz. Information on the general shape, head/body proportions, eye coloration and plumage characteristics of owl species have been obtained from the *Collins Bird Guide*^24^, arguably one of the most authoritative books available for the identification of European bird species^25^. Curiously enough, the owls (Order Strigiformes) depicted in the aforementioned guide, and actually in every bird guide, are the only species depicted frontally, with the two eyes staring at the reader. It seems that humans, from the very first representation of owls in parietal art (Chauvet Cave, France), reserve for the owls, and just the owls among birds, a frontal representation^24^ to reinforce and make unmistakable their owl being. We have placed particular importance to an aspect overlooked by previous researchers: the holes on the “heads” of the “2D” flat plaques, and the lack of them on the idols carved on “3D” substrates such as bones, and thus having a volume. We hypothesize that two holes make no sense to pass a string, as previously proposed despite their heavy weight (more than 300 g on average^2^), and the fact that they typically have no use-wear traces, as remarked by Lillios^2^ and Thomas et al.^3^. Instead, we suggest that the holes were used to insert actual feathers to simulate ear tufts (Fig. 3), of the type present in only two local species, the long-eared owl (*Asio otus*) and the eagle owl (*Bubo bubo*)*.* To outline the silhouette of owl tufts on thin slate plaques would have been very difficult, as they would easily break. The use of actual feathers would solve the problem. At the base of the feather, the rachis, or central shaft, gives way to the hollow tubular *calamus* (e.g., Horváth et al.^26^). The calamus, or quill, with a rounded section of about 2–3 mm (diameter) in an (medium sized) owl’s flight feather, is the part that may be easily inserted in the hole(s) of the plaque. It may be twisted so that the remaining of the feather stays up and aligned with the plaque. Current and historic ethnographic evidence indicates that different cultures have carved figurines, often anthropomorphic, in which actual feathers have been inserted on top of the head. To gain further support for this interpretation, we have tested design types against number of holes, assuming that the most owl-like plaques should be the ones having two holes, whereas the less-owl-like plaques should tend to have just one or none. We have restricted the statistics to testing the number of holes in relation to the degree of owliness in a sample of plaques contained in the catalogue created and maintained by Lillios^27^. We generated a dataset of 100 plaques, including zoomorphic and non-zoomorphic in similar proportions, and recorded presence/absence of six morphologic traits present in actual owls (i.e., two eyes, feathery ventral area, rostral marks, facial disk, bill and wings). The presence of each one of these characters was considered as one point (1) in our owl score, with its absence counting as zero (0). This allowed us to assign a total owl score for each plaque, ranging from 0 to 6. We related this score with the number of perforations through a Kruskal–Wallis test.

To gain support to our hypothesis that the slate plaques and some related objects were children dolls or toys of little ritual value that may have been crafted for young people and not necessarily by experienced artisans of the community, we establish a comparison with owls as seen by contemporary children, taking therefore an actualistic approach (see, e.g., Blanco et al.^28^). We assume that the differences in the craftsmanship of the plaques may well result from differences in the technical skill of the engravers associated to age and experience, as we see today in owl drawings depicted by children of different ages. With this in mind, we extracted owl images using Google^29^. We specifically searched for images drawn by children 4 to 13 years-old. We selected 100 drawings in which the age of the children was specified, and generated a score for each image based on the presence/absence of the same six owl traits (i.e., two eyes, feathery ventral area, rostral marks, facial disk, bill and wings) that we described above to evaluate the owliness of the slate plaques. Every recognizable trait adds one point, allowing us to obtain a complexity score for each drawing. We related the score with the age of the children through a Spearman correlation.

## Results

We clearly see owls depicted in some of the engraved plaques, particularly the ones previously (and aptly) categorized as zoomorphic or biomorphic^2,27^. We specifically refer to those plaques with a trapezoidal shape, in which the head follows the contour of the body, with two large eyes within facial disks. In the alternating pattern of dark and light triangles under the head we recognize the camouflaged plumaged characteristic of the ventral area of most owl species (Fig. 2). This way of representing plumage patterns in birds is still used by contemporary artists, such as American modernist painter Charley Harper, who sketched numerous owls using just a few lines^30^, and defined his style as “minimal realism” (see also Fig. 2S). In the particular subset of plaques made of sandstone, so far interpreted as anthropomorphic figures having engraved arms and hands, we see the owl’s wings, and a representation of the tip of the primary feathers. One case in point is a plaque with curved arms terminated in seven “fingers” (see Fig. 1G). This object is best interpreted as an owl with folded flight feathers. Another outstanding example is a slate plaque with two bird feet engraved at the base^31^, with three and four forward toes, respectively (Fig. 1B and, for more detail, Fig. 3S). This is an exceptional piece, as it is totally comparable to owl drawings made by modern children (Fig. 5).

As additional support for our owl interpretation, we have used Google Lens, an image recognition technology developed by Google that uses visual analysis, on the picture of the Valencina slate plaque in Fig. 2. Out of 60 coincidental images offered by the system, 27 were manufactured owls, 10 were Chalcolithic plaques or replicas, and the remaining (and offered last) different textiles showing geometric motifs. Therefore, a blind algorithm based on neural networks publicly and freely available to anyone with internet access, also identifies an owl where we see it engraved on a slate plaque. The use of Google Images as a tool in Ecology and Evolution was proposed years ago^29^.

### European owls

There were seven owl species potentially distributed in southern Iberia during the Copper Age, the same ones living in the area today^24^. These species greatly differ in size (Table 1), ranging from the scops owl (*Otus scops*, 92 g) to the Eurasian eagle owl (*Bubo bubo*, 2.7 kg). Only two of those owl species have remarkable ear tufts (i.e., the eagle and the long-eared owl [*Asio otus*]), whereas the scops and the short-eared owl (*Asio flammeus*) have just a hint of tufts and often they are hidden altogether. The remaining species have no tufts at all. Two species have dark eyes (i.e., the barn owl [*Tyto alba*] and the tawny owl [*Strix aluco*]), whereas the remaining species have bright yellow to orange irides contrasting with the black pupil at the center. Even though all species possess highly cryptic plumage patterns (except the frontally all-white barn owl), different plumage patterns may be ascertained. Both eagle and long-eared owl, for instance, have dark strikes in the frontal plumage with lateral spikes. In the most figurative plaques we interpret the eyes has having a central pupil and a differently colored iris. The lines protruding from the iris, described by some scholars as sun rays^3^, we interpret as the highly modified feathers of the owl’s facial disk, that are disposed radially around the eye (Fig. 3).

**Table 1:** Presence/absence of tufts and eye coloration, morphometrics, population size and ratio (in relation to population size of less common species, *Bubo bubo*) in Europe, of the owls present in southern Iberia.

Species	Ear tufts	Eye colour	Body mass (g)	Length (cm)	Population size Europe	Population ratio
*Strix aluco*	No	All dark	380–800	37–43	1,520,000	33.1
*Asio otus*	Long	Orange-black pupil	250	31–37	788,000	17.2
*Athene noctua*	No	Yellow-black pupil	170	23–27	1,560,000	33.9
*Tyto alba*	No	All dark	430–620	33–39	239,000	5.2
*Otus scops*	Short	Yellow-black pupil	92	19–21	566,000	12.3
*Asio flammeus*	Short	Yellow-black pupil	200–450	33–40	215,000	4.6
*Bubo bubo*	Long	Orange-black pupil	2700	59–73	45,900	1.0

Body length from Svensson et al.^24^. Population sizes as in IUCN Red List^65^.

**Figure 4:**
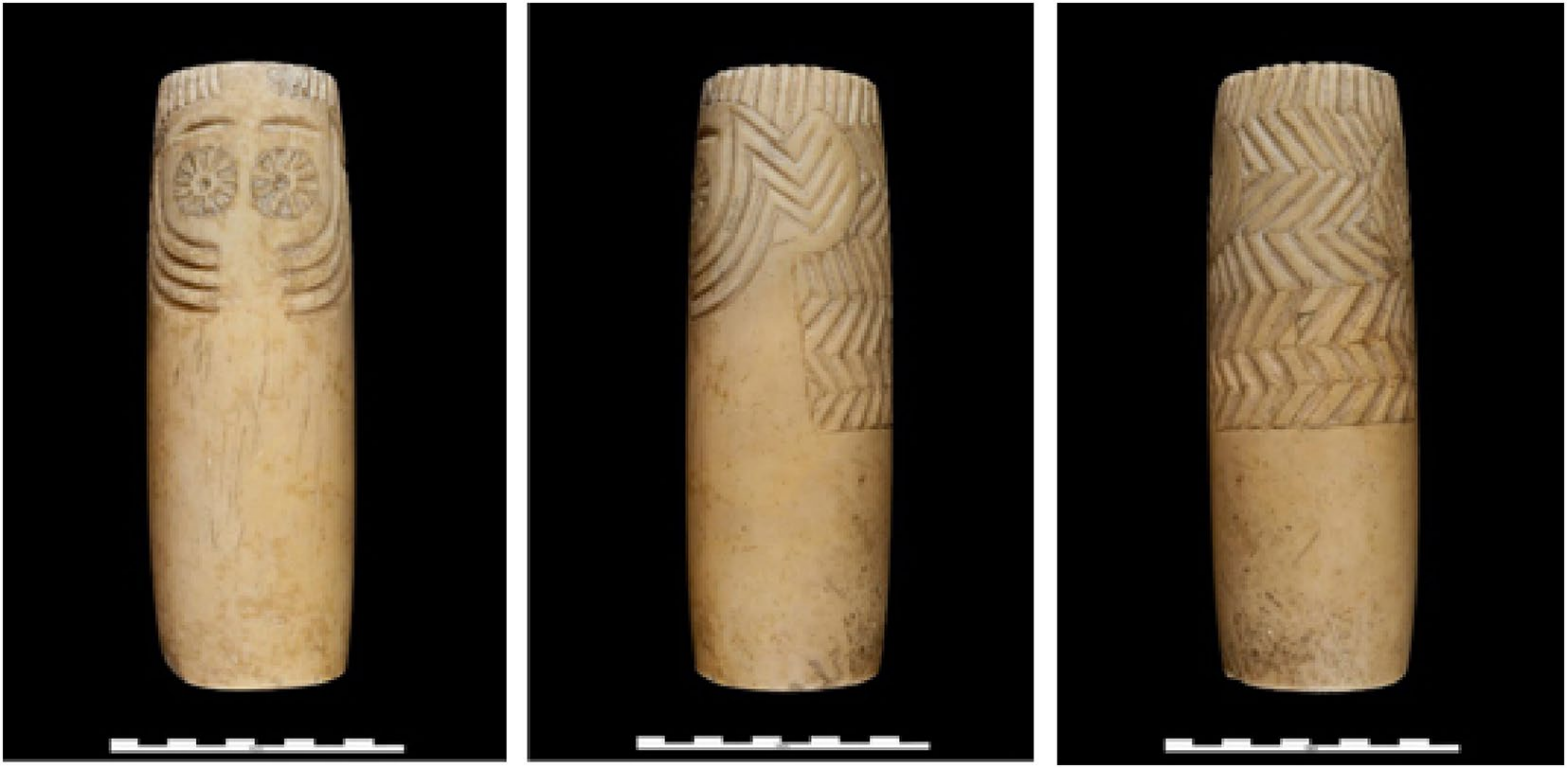
Oculated idol carved in alabaster known as *Ídolo de Extremadura* (unknown provenance, MAN, Madrid, 20572), and perhaps modelled after a long-eared owl. Tufts were carved on the sides and back of the head. The zigzagging pattern in the back, interpreted as long hair, may also be described as the camouflaged plumage pattern in the dorsal area of an owl.

We suggest that two of the most common and widely distributed species (Table 1), the little owl (*Athene noctua*) and the long-eared owl (*Asio otus*), were the model for a majority of the owl-like plaques. For the plaques with dual perforations, and if they were used for inserting actual feathers, we favor the idea that long-eared owls were the most probable model (Fig. 3). This ubiquitous and abundant species, mainly living in semi-open habitats that may have been commonplace around early human settlements, is the quintessential owl, with large and contrasting ear tufts, facial disks, rostral marks, bright orange irides with a dark central pupil, and an all-over patterned plumage pattern. The specific behavior of these owls may also matter. They are the most social of all owls in the region, forming communal winter roosts, sometimes with dozens of individuals sharing the same tree or small grove^32^. The long-eared owl may also be the model of some cylindrical types of idols, contemporary of the slate plaques but more abundant in southeastern Spain, and other places were natural slates are lacking^6^. The idol in Fig. 4 presents carved “owl tufts” on the sides of the head. The barn owl was the model for the head of a Copper Age figurine found at Badajoz province (Fig. 4S) and described so far as an anthropomorphic idol^13^.

### Perforations in the plaques

We can observe that higher owl scores, and thus plaques with more owl characteristics, are linked, on average, with a higher number of perforations (Kruskal–Wallis test, *z* = 22.458, df = 3, *P* < 0.001, *n* = 100, Fig. 6a). This indicates that the increase in the number of perforations runs in parallel to the complexity of the representation and could be related with the insertion of actual feathers depending on their availability to increase a mimetic representation, as previously hypothesized, even though feathers simulating owl ears could be properly inserted using a single piercing. Those feathers may have been recovered as moulted feathers, as they are shed once a year by wild birds and are easily found on the ground, or were procured from dead or hunted specimens.

### Craftsmanship of the plaques

**Figure 5:**
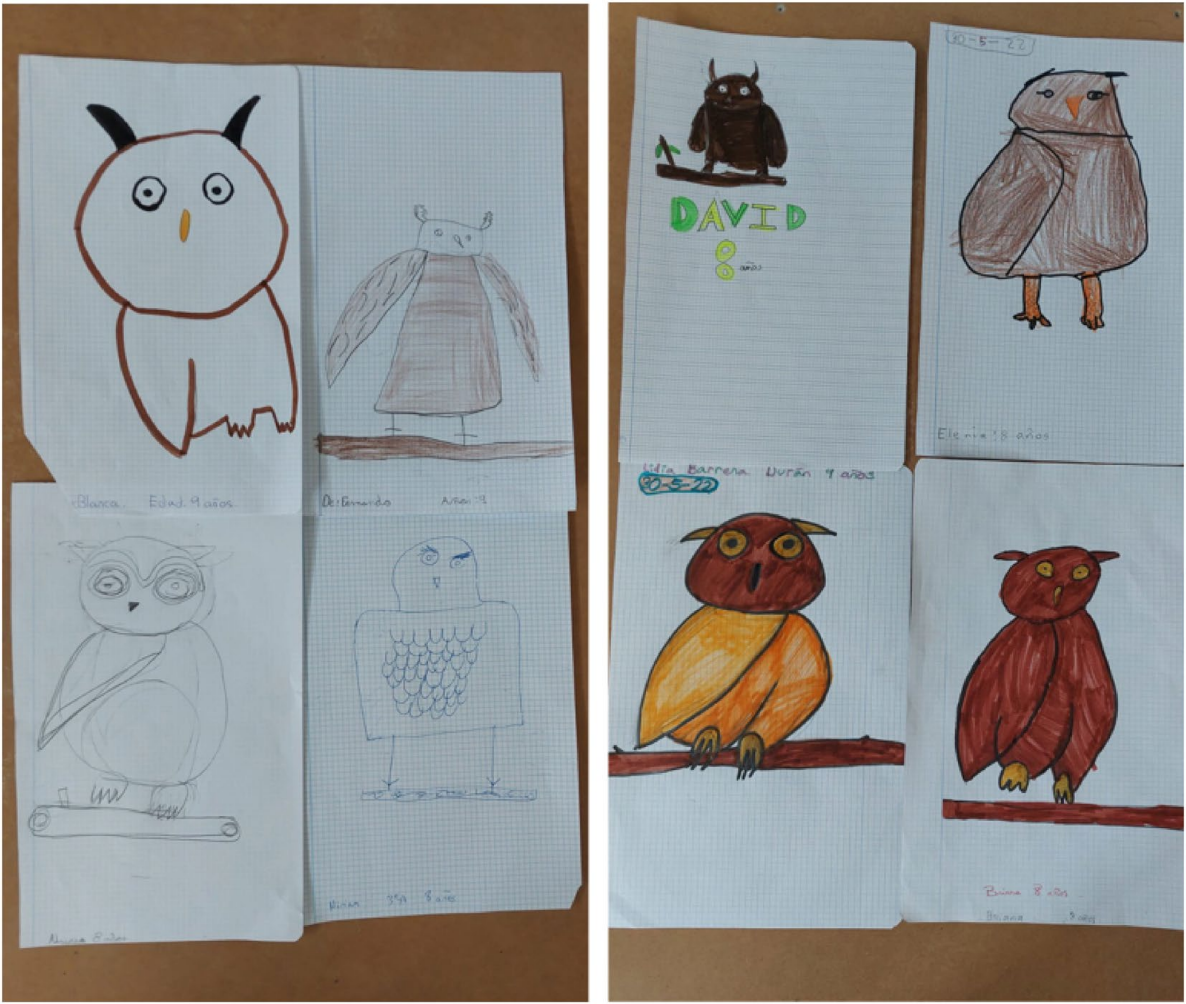
Owls painted by 8-years old children from a primary school in southwestern Spain in 2022. The students were asked by their teacher to sketch an “owl” in less than 20 min, with no further instructions. The resulting drawings have in common a frontal view with frontally placed round eyes with differentiated iris and pupil. In the sample, all owls but one spot ear tufts. The use of Google Lens on this mosaic figure shows as coincidental 60 images of owls drawn by infants or as owl silhouettes to be colored. No other objects have been identified by the algorithm.

**Figure 6:**
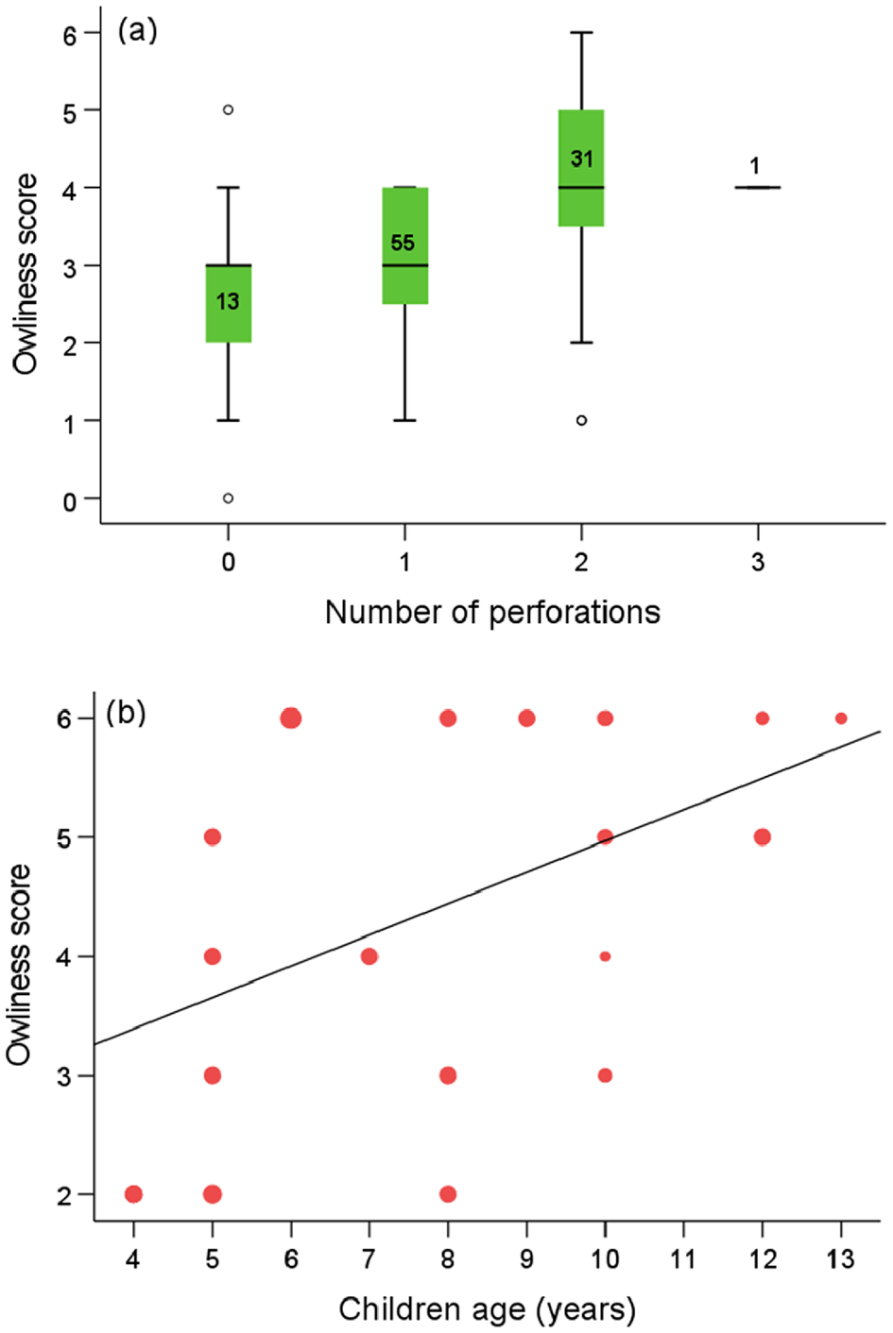
(**a**) Boxplot showing the relationships between the number of perforations and owliness score of a random sample of slate plaques. Numbers inside the box represent sample sizes. (**b**) Relationship between children age and owliness score in a random sample of 100 drawings. The increasing size of multiple markers represents 1, 2, 4, 5, 6, 7 and 12 cases, respectively. Least squares regression line of the correlation is shown for graphical representation of trend.

The casual style of the plaques led us to hypothesize that they may have been crafted by the young themselves, or by semi-skilled members of the community, as recognized by Thomas et al.^3^ for the slate plaques they studied and replicated (and contrasting with the highly skilled makers of exquisitely worked pieces of jewelry also found in Copper Age funerary monuments^33^). Slate exfoliates easily in sheets and it does not need to be mined underground nor carved with special tools, or put in an oven as with metal melting or pottery firing^16,17,22^. Slate sheets are practically blank canvases ready to be used if the intention is to engrave them. In addition, when the stone is carved, it has the peculiarity of alternating its natural black color with the white of the lines that have been engraved, a characteristic that facilitates the imitation of the cryptic plumage of the owls. As with Thomas et al.^34^, we expected differences in the craftsmanship of the plaques, that may well result from differences in the technical skill of the engravers associated to age and experience, as demonstrated for prehispanic pottery makers in the American Southwest^16,17,22^. Our actualistic approach, in which we resorted to a comparison with owl drawings by contemporary children of different ages clearly reveals two facts (see Fig. 5): (a) owls are always drawn with the head situated frontally, with the owl’s eyes staring at the observer (as if there were no other ways to depict an owl), and (b) there is a progression related to age in the owliness of the depictions, with more and more owl characters added by older children (Spearman rank correlation coefficient, *r*_*s*_ = 0.45_,_ *P* < 0.001, *n* = 100, Fig. 6b). The latter may help explain at least partially why there are so many plaque types, and why some are more evocative of owls than others.

It is clear to us that a stone burin is not a crayon, and slate is not paper. However, Thomas et al.^34^, who conducted replication experiments of Chalcolithic plaques with university students, considered slate as an “easily worked material, malleable and highly responsive to engraving tools of different hardnesses”. The engraving of the plaques varies in execution, including some rough models with few contorted lines (as plaque f in Fig. 1) and others more elaborated (as plaque a in Fig. 1).

## Discussion

### Owls in human culture

Owls are unique among birds due to their large heads and frontally placed eyes^23^. Only for this, they are the most anthropomorphic of all birds, and perhaps of all animals if we exclude our closest relatives, the apes. A consequence of the owls’ human-like shape is that they are depicted differently to all other birds, and in fact other animals, from the dawn of art in Paleolithic parietal manifestations to today. If animals are systematically depicted laterally on a side view, as with contemporary animal identification guides, owls are depicted frontally, or at least with the head turned to show their two eyes. This is so in parietal engravings as old as 30,000 years during the Aurignacian period^23^, but also in the Classical Greek period with the representation of the little owl in coins and ceramics, as the accompanying owl of the goddess Athena, as well as in mosaics (i.e., the owl in the mosaic of the Domus of the Birds in Italica, Spain). Owls have been represented frontally in parietal panels by different peoples and epochs worldwide, including Australia, southern Africa, the New World, or the Levant art of eastern and southern Iberia in the Chalcolithic^35^. Owls are so entwined in human culture, that, if asked to do so, both urban and rural children attending elementary school anywhere in the world would represent one easily and recognizably as a large-headed bird looking at the observer with two big eyes^23,36^. In this respect, owls are—and possibly were in a distant past—as iconic and familiar as a cow or a horse. Of all bird families, the owls and the diurnal birds of prey are perhaps the only ones that every human will recognize as culturally significant^37^. A survey of the bird shapes most preferred by humans, revealed that the shorter the neck and the bigger the eyes the most attractive resulted the species, with owls ranking among the most attractive bird types^38^.

Since the discovery of the first engraved slate plaques, they were ascribed a profound symbolic and ritual purpose^7^. Despite its simplicity, their artistry was also praised and readily compared to other crafts produced in other parts of the Old World, from the Iberian Peninsula to Mesopotamia^9,10^. Gimbutas^10^ visually described the plaques as “owl goddesses on a trapezoidal plaque of schist”, also carved on bones or in pottery. After Gimbutas, only the goddess interpretation remained, and the owl model was forgotten.

### The making of the plaques

Lillios^2^ described experiments aimed at replicating engraved plaques using tools at the disposal of the ancient engravers. Later, Thomas et al.^3,34^ also reported the process of replicating plaques by university students with no previous artistic background. A modern experimenter would produce a finished plaque in about 3.5 h. This is important to remark, as it contrasts in terms of labour and complexity with other technological achievements by the same Chalcolithic peoples. Polished tools, such as handaxes, had been in place for millennia, and the beaker ceramics characteristic of the Iberian Chalcolithic included similar zigzagging patterns. However, the latter involved more manufacturing steps and the mastering of firing techniques. In other words, coeval everyday life objects predated the technology and styles of the plaques. In addition, the Chalcolithic culture is characterized by the erection of megaliths, composed by large and heavy stones weighing sometimes more than 150 tons. Some of these large stones were moved across relatively large distances^33^. It would have taken the coordinated participation of numerous people along protracted periods of time to build a single tholos, for instance. We establish this comparison to suggest that perhaps the social role, complexity, and value of the slate plaques may have been misunderstood. Only local materials were needed to procure the plaque itself (i.e., slate), to grind and polish it (e.g., granite and leather) and to engrave it (e.g., a flint, quartz or copper burin)^3^. In fact, there is no clear evidence of exchange or commerce with the plaques, as it was the case with luxurious items whose raw materials originated in distant places^33^.

Object manufacture involves the choice of a material for its accessibility and physical characteristics. Also, the use of tools in the framework of learning and developing an activity, i.e. stone carving, fundamental in the structuring of megalithic societies, as well as a shared social framework in which play and the toys acquire their playful meaning, since it should not be forgotten that it is play that gives meaning to the toy^14^, contributing to strengthening the bonds among group members. Considering the abundant raw material used in their making, plus how fast they could have been produced, and the importance of stone carving in other social contexts, such as the construction of megalithic tombs, we suggest their social value may have been different than that of other pieces crafted concurrently but using precious materials or rare rocks including gold, elephant ivory or rock crystal, which originated in distant areas sometimes hundreds of kilometers away from the site of discovery^39^. In the latter case, as with the exquisitely crafted rock crystal arrowheads found inside the Montelirio tholos, of the Valencina Copper Age settlement, the participation of highly skilled and experienced artisans is inferred. Curiously enough, researchers remarked in surprise that no slate plaques or “idols” were found among the hundreds of luxurious items found at Montelirio, in a clearly symbolic and ritual context^33^.

Competent engravers, perhaps adults or adolescents, may have initiated novice children. Whether this learning process took place in the household or in other production contexts is unknown; as of today, only one possible plaque workshop has been found with plaques at different stages of production (Águas Frias, Evora, Portugal)^3^. To determine the motor skills and mental abilities needed to use a stone burin on slate is out of the scope of this investigation, but it may be experimented in the future with children of different ages, even if the cultural context today is completely different to the distant past. The children of stone carves grew in an environment where they had first the chance to learn by direct observation and imitation, and later by collaboration with experienced carvers in increasingly difficult tasks. It is already established that children aged 7–9 years have the ability to recognize patterns and styles, as well as to draw them using paintbrushes^16,17,22^. We leave open the question on the actual earlier age of child participation in the making of slate plaques, as we have no current ethnographic examples to compare to.

### Idols or toys

We concur with Maicas^18,40^ that it cannot be discarded that some “idols” were in fact objects with just a recreational value, literally dolls that may have entertained both their makers and younger members of the community in playful activities or as learning activities^41^. We propose that some plaques may have been decorated with actual feathers. The use of feathers as ornaments is widespread in multiple ethnographic examples around the world, and has even been inferred even for Neanderthals predating the arrival of *Homo sapiens* in Europe^42^. The interaction of human-gatherers with birds of prey in particular is not one of hunting as food items, as evidenced by numerous ethnographic examples^43,44^, and includes the keeping of pets^14,45^. Some of the plaques were painted, and some of the idols may have been dressed with textiles^40^, reinforcing the idea that at least some plaques functioned as dolls. In multiple ethnographic examples in both the Old and the New Worlds, dolls are made for children by adults or, very often, by the children themselves^14,45^.

The fact that many plaques have been found in funerary contexts may reflect that they were at some point used as a tribute to the deceased. Offering plaques would thus be part of a community ritual. This may also indicate that toy or doll offering may have been a way in which the younger members of the group participated in the funerary rituals practiced by adults. Given the highly mimetic character of games, toys (or pieces manufactured by youngsters in any case) could have been offered when such rituals were imitated by the children as part of some of their games. After all, everyday life objects such as pottery and polished tools have also been found in burial contexts. The slate plaques of the Chalcolithic predate one millennium the set of toys of the Siberian Bronze Age made after animal models including birds. They were supposed to be used by children, and were finally deposited at baby graves, as if they had to remain with their deceased owners^21^.

### Owls as a familiar presence

At this point, we can ask why owls may have been used a models for the first dolls/toys ever made in Europe. Special relationships with owls are evidenced even before the onset of the Neolithic. The two older examples of bird parietal art have been found in French caves: the tufted owl engraved at Chauvet, and a family of three snowy owls (*Nyctea scandiaca*) at the cave of Trois-Fréres^23^. At the cave of Bourrouilla (also in France), the skeletal remains of 53 snowy owls highly modified by humans were found in levels of the Upper Paleolithic^46^. The dark tent tradition, a ritual shared by indigenous peoples in circumpolar northern regions, is performed at night by a shaman emitting animal calls, including owls, and it may have been introduced into North America by Siberian peoples about 5000 years ago^47^.

Owls, as we said above, are unique in shape and appearance resembling little humans. But behaviorally they are also special in many respects. First, a majority of species may live very close to people. All seven species occurring in southwestern Iberia are found in urban areas today. This may have been so in the past too. The long-eared owl, that we proposed as the probable model for the most realistic plaques, are not generally afraid of humans, and are common urban dwellers, possibly since the inception of the first settlements^48^. They are avid consumers of mice and voles and they were possibly identified as helpers and/or benefactors of the early farmers cultivating and storing cereals, well before the introduction of rodent-killing domestic cats in the Iberian Peninsula by either Phoenicians^49^ or later the Romans^50^. Two ceramic vessels found in a megalithic tomb in the Netherlands dated about 5,000 years BP had been decorated using the head of the femur of a long-eared owl, indicating that this species was also familiar, and possibly significant, to other peoples in the Chalcolithic outside Iberia^51^.

### Play and objects to play

Children´s object play, and the objects themselves, has been disregarded in the archaeological literature until recently^14,19,45,52–55^, even though object play is ubiquitous in both tribal and modern societies. Its importance in human evolution and as a driver of innovation is gaining momentum^52–55^ In fact, object play is not exclusive of humans, and it has even been described in birds^56^. However, play based on pretending by employing our capacity for meta-representation of objects, phenomena or people, a cognitive capacity that motivates both playful and ritualistic behaviors, is unique to humans^57–60^. At this point, we may ask why previous authors interpreted ritual objects (idols) where we see recreational and learning objects. Dissanayake^53,61^ pointed out how blurred are the boundaries between art, ritual and play. For this author, play is a social, voluntary and spontaneous activity. Its most notable characteristic is its metaphorical capacity, being a behavior based on exaggeration and imitation: one thing becomes another, being able to express itself in different ways. Imitation becomes a simulation where the referential limits of the initial phenomenon are dissolved. The initiation of learning skills to produce functional objects, which were often decorated, has attracted the attention of scholars^41,62^. P. Crown^16,17,22^ has pointed out, for instance, that learning to make pottery begins in childhood in pottery-producing societies worldwide. Southwestern Pueblo children began to learn to make pottery as early as age five^16^.

Play offers the child an activity in which he or she can learn the possibilities of ontological mutability necessary for all mimetic behavior^56^. Thus, ludic behavior can be both the consequence and the precursor of other more serious behaviors, among them, the aesthetic and the ritual, with whom he also shares his ability to manage stress^58^. These capacities and emotions were surely present in our ancestors^59^. We are exactly the same species, even if culturally we have greatly evolved in the last few millennia. From this point of view, we can define the owl-like objects as the product of a playful simulation of reality, being metaphorical objects that imitate and exaggerate a specifically summoned phenomenon, that may have to do with frequent encounters with actual owls, creatures of the night with salient anthropomorphic features. In their social role, these objects had to behave as active patients with respect to the agency exercised by another, that is, they were treated by their possessors as an "alter ego and a social other," as proposed by Gell^63^.

We wish to remark that our hypothesis that the slate plaques of the Iberian Peninsula in the Chalcolithic were toys inspired on owls which may have had, at least originally, a recreational use, is based on the transcultural fascination of humans by owls since time immemorial^23^, in turn due to their peculiar anthropomorphism that predispose us to pay attention to them. Additionally, applying Ockham’s Razor, our hypothesis is simpler that the alternative of resorting to a complex symbolic world with fertility goddesses represented by idols^9,10,13,64^, or heraldic mnemonic devices as proposed by Lillios^2^, of which there are no proofs anyway. Why the manufacture of stone toys was discontinued about 5000 years ago may have to do with the advent of new technologies. If stone toys were made at the end of the stone age, metal tools in subsequent periods surely made easier the carving of wood figurines, which would hardly leave any traces in the archaeological records. Similarly, skin or textile pieces would disintegrate quite rapidly. Therefore, owl-like objects made in stone provide perhaps one of the few glimpses to childhood behaviour in the archaeological record of ancient European societies^14,19,55^.

## Supplementary Information

Supplementary Information.

## Data Availability

The datasets used and/or analyzed during the current study available from the corresponding author on reasonable request.
