# Supplementary material for: Owl-like plaques of the Copper Age and the involvement of children
Source: Sci Rep. 2022 Dec 1;12:19227. doi: 10.1038/s41598-022-23530-0 (PMC9715531; doi:10.1038/s41598-022-23530-0)
Supplement: Supplementary file 1 — Supplementary Information. [file 41598_2022_23530_MOESM1_ESM.docx]

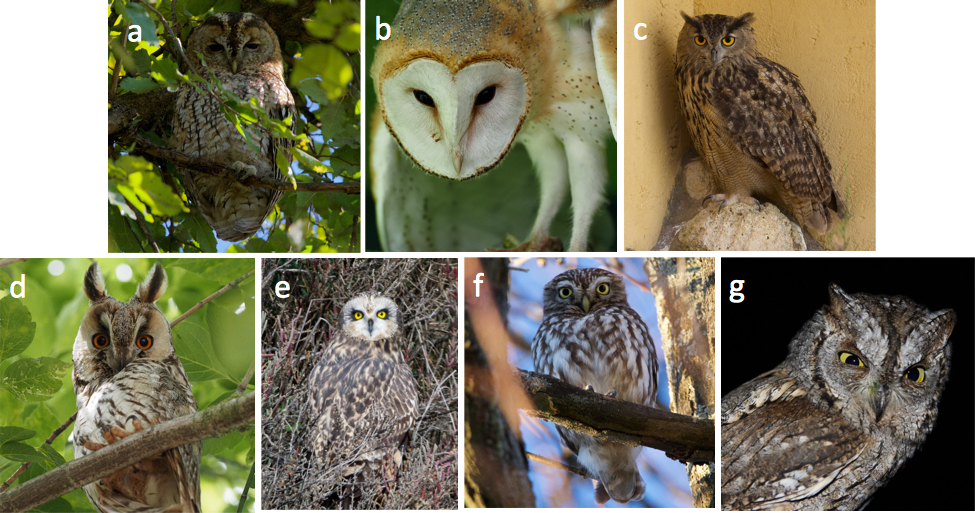


Fig 1S. All 7 owl species distributed in the southern portion of the Iberian Peninsula: (a) tawny owl (*Strix aluco*), (b) barn owl (*Tyto alba*), (c) eagle owl (*Bubo bubo*), (d) long-eared owl (*Asio otus*), (e) short-eared owl (*Asio flammeus*), (f) little owl (*Athene noctua*), (g) *s*cops owl (*Otus scops*). Credits: all pictures by Juan J. Negro, except scops owl by Pedro Molina Holgado.


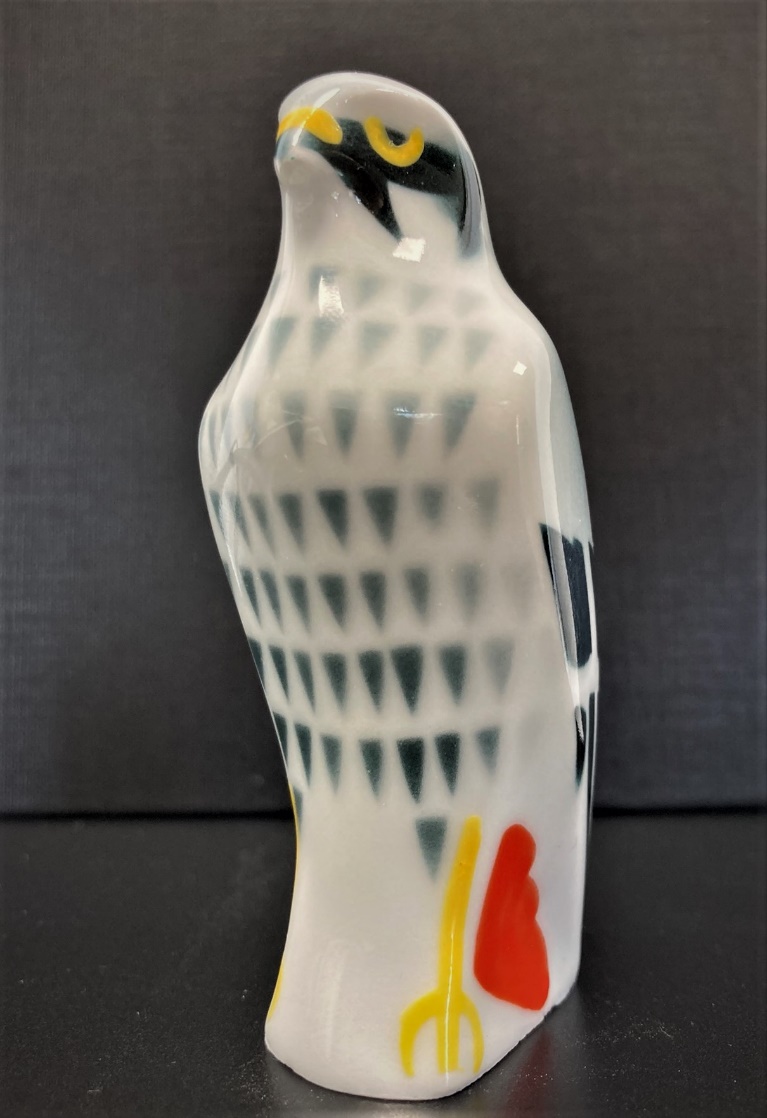


Fig. 2S. Ceramic figure sold by the Sargadelos company, located in northern Spain (<https://sargadelos.com/es/naturaleza-y-fauna/16109-halcon-pq.html>), accessed 27 June 2022). It is called “small falcon” and represents a bird of prey with inverted dark triangles in the frontal area used to mimic the striped breast and belly of a true falcon, as with the Chalcolitic owls. This is a 20^th^ century design still on sale in the catalogue. The artist was possibly unaware of the existence of the Chalcolithic slate plaques. Credit: Juan J. Negro.


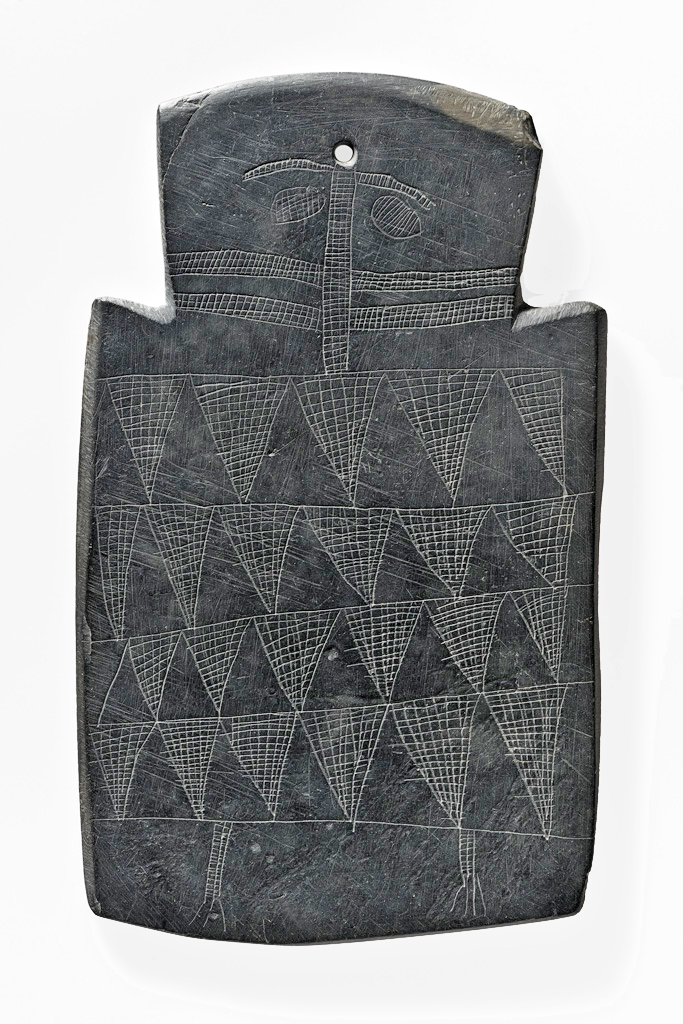


Fig. 3S. Engraved plaque with “bird feet”. This is an exceptional piece, even if minimalistic in style, as it depicts very clearly an owl with feet at the base and two eyes in the face, plus the camouflaged plumage pattern represented by alternating triangles. Found at Anta do Curral da Antinha, Portugal, and deposited at Museu Nacional de Arqueologia de Portugal, No. 2003.37.1, Lisboa.


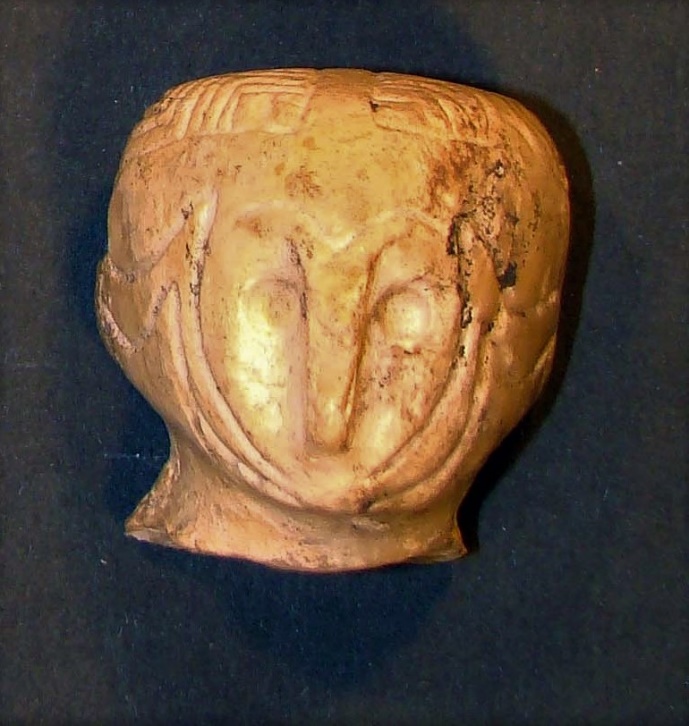

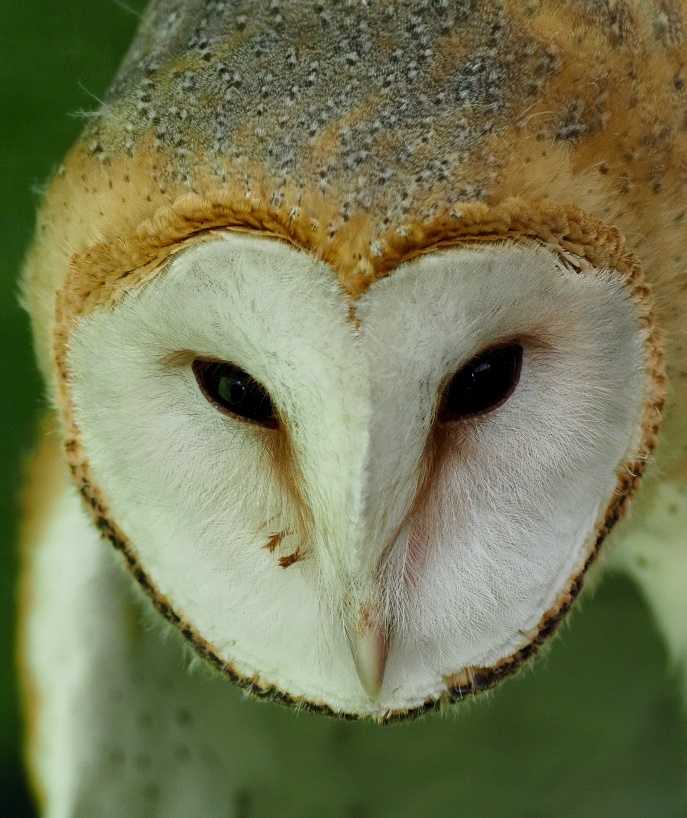


Fig 4S. The idol head from the Copper Age, found at La Pijotilla site (Badajoz Province) and deposited at Museo Arqueológico Provincial de Badajoz, was modelled after the face of a Barn Owl (*Tyto alba*). The bilobed, or heart-shaped, face with two round eyes in the upper third is unique to this owl species, and no other bird or animal resemble it. Credits: figurine photographed by Guillermo Kurtz (Museo Arqueológico Provincial de Badajoz). Barn owl by Juan J. Negro.

**Annex 1. Webpages accessed to score child-painted owls:**

1: <https://www.pinterest.es/pin/my-6-year-olds-drawing-of-an-owl--406731410076394942/>

2: <https://www.dreamstime.com/drawing-owl-child-years-old-colored-pencils-isolated-children-s-creativity-paper-image120230458>

3: <https://www.pinterest.es/pin/347058715018705557/>

4-7: <https://www.barnowltrust.org.uk/owl-facts-for-kids/kids-owl-pictures/>

8: <https://glenviewschoolofarts.com/ages-68>

9: <https://maclachlan.ca/mac-programs/ib-pyp-pk-6/>

10: <https://www.poolacademy.co.uk/latest-news-1/2020/6/2/year-6-use-half-term-to-write-letters>

11: <https://mouhoxlab.wordpress.com/tag/children-drawing/>

12-17: <https://artlessonsforkids.me/2011/11/25/teach-how-to-draw-gorgeous-owls-in-grade-one/>

18-22: <http://artwithmrssmith.blogspot.com/2012/10/owls-for-fall.html?m=0>

23-34: <https://www.helloartstudio.com/sg-september-2018-charcoal-owls>

35-36: <https://frugalfun4boys.com/exploring-nature-with-kids-by-observing-and-sketching/>

37-39: <https://kinderart.com/art-lessons/drawing/owl-reflections/>

40: <https://www.vecteezy.com/vector-art/2752870-outline-drawing-with-funny-hedgehog-owl-elephant-fish-creative-childish-texture-in-handmade-style-great-for-fabric-textile-wrapping-paper-decor-design-in-a-children-s-style>

41-43: <https://laclassedemelusine.fr/?utm_source=melusicanalblog?&utm_medium=alert2redirect?&s=Tuto%20de%20la%20chouette%20en%20dessin%20dirig%C3%A9>

44-55: https://kknews.cc/baby/a2ba83x.html

56-61: <https://kknews.cc/culture/j4pkn5y.html>

62: <https://www.google.com/url?sa=i&url=https%3A%2F%2Fhandmadebase.com%2Far%2Fsimple-image-owls-for-children-ful%2F&psig=AOvVaw2SzQSEJnf14iBKX9wJWkbX&ust=1652718592747000&source=images&cd=vfe&ved=2ahUKEwjpjazW9uH3AhULlRoKHQafANQQr4kDegUIARDHAQ>

63-70: <https://kgoo.blogspot.com/2013/11/blog-post_24.html>

71: <https://roayatwatneg.com/wp-content/uploads/2021/07/IMG_20210704_223759-scaled.jpg>

72: <https://artprojectsforkids.org/how-to-draw-an-easy-owl/>

73-76: <https://budivel.ru/hi/pol/kak-ukrasit-divannye-podushki-svoimi-rukami-kak-sshit-divannuyu-podushku.html>

77-80: <https://www.google.com/url?sa=i&url=https%3A%2F%2Fwww.soescola.com%2F2018%2F01%2Fdecoracao-com-tema-corujinha.html&psig=AOvVaw2Uys6UyzABMUhMuyz9kD60&ust=1652719273871000&source=images&cd=vfe&ved=0CA0QjhxqFwoTCPj976r54fcCFQAAAAAdAAAAABAE>

81-85: <https://br.freepik.com/vetores-premium/desenhar-ilustracao-vetorial-para-colorir-pagina-de-corujas-fofas-com-conceito-de-volta-as-aulas-estilo-de-desenho-animado-doodle_16983912.htm>

86-96: <https://atividadespedagogicas.net/2019/05/desenhos-de-corujas-para-colorir.html>

97-104: https://www.google.com/url?sa=i&url=https%3A%2F%2Fwww.pinterest.dk%2Fjettepihljensen%2Ftegning-ugler%2F&psig=AOvVaw31h6A1VGr4H2_JZJvOno-9&ust=1652719584465000&source=images&cd=vfe&ved=0CA0QjhxqFwoTCNif3bT64fcCFQAAAAAdAAAAABAD
